# To fail is human: remediating remediation in medical education

**DOI:** 10.1007/s40037-017-0385-6

**Published:** 2017-10-25

**Authors:** Adina Kalet, Calvin L. Chou, Rachel H. Ellaway

**Affiliations:** 1 Program on Medical Education Innovation and Scholarship, New York University School of Medicine, New York, USA; 2 Department of Clinical Medicine, Academy of Medical Educators University of California, San Francisco, USA; 3 Office of Health and Medical Education Scholarship at the Cumming School of Medicine, University of Calgary, Calgary, Canada

**Keywords:** Remediation, Medical education, Competency based medical education

## Abstract

**Introduction:**

Remediating failing medical learners has traditionally been a craft activity responding to individual learner and remediator circumstances. Although there have been moves towards more systematic approaches to remediation (at least at the institutional level), these changes have tended to focus on due process and defensibility rather than on educational principles. As remediation practice evolves, there is a growing need for common theoretical and systems-based perspectives to guide this work.

**Methods:**

This paper steps back from the practicalities of remediation practice to take a critical systems perspective on remediation in contemporary medical education. In doing so, the authors acknowledge the complex interactions between institutional, professional, and societal forces that are both facilitators of and barriers to effective remediation practices.

**Results:**

The authors propose a model that situates remediation within the contexts of society as a whole, the medical profession, and medical education institutions. They also outline a number of recommendations to constructively align remediation principles and practices, support a continuum of remediation practices, destigmatize remediation, and develop institutional communities of practice in remediation.

**Discussion:**

Medical educators must embrace a responsible and accountable systems-level approach to remediation if they are to meet their obligations to provide a safe and effective physician workforce.

## Introduction

Remediating failing learners is an essential part of medical education but one that can be taxing to the remediated, their remediators, and to health and educational systems in general [1]. Although there has been a growing focus on remediation in the medical education literature [2], it has tended to be on the mechanics of remediation or on matters of due process and defensibility. What is missing is a more theoretical basis for remediation, such as how remediation practices do and should intersect with their parent medical education systems. It is time to address these issues by remediating current practices and perspectives on remediation in medical education.

In 2014, at the American Association of Medical Colleges annual meeting, we conducted a symposium entitled *Remediation as an Emerging Issue in Medical Education*. From this, we committed to produce two commentaries that emerged from the rich discussion. Central to our discussion was a need to connect remediation with broader debates in and perspectives on medical education. To that end, in our first paper we modelled remediation as a zone of practice in medical education with particular rules and expectations that sit alongside other zones of practice, each with its own rules and expectations [3]. In this complementary paper we explore the concepts and practices of remediation in the broader contexts of medical education, and we consider the inescapably contextual nature of remediation. We build a systems-level theory for remediation policy and practice from this work, and make recommendations for future practice.

Remediation in medical education is ‘*the act of facilitating a correction for trainees who started out on the journey toward becoming a physician but have moved off course*’ [4]. Remediation is a necessary component of medical education, not only for the sake of struggling learners, but also as a way of assuring the quality of the physician workforce. In ideal circumstances, remediation should involve a series of prescribed and officially sanctioned episodes of additional corrective training and monitoring, ending with an assessment of whether the learner has met the predetermined set of remediation goals [5]. The form and depth of each episode of remediation should reflect the learner’s deficits, ranging from support and correction while in service (if the remediated deficit or practice context is low risk), through full retraining with reduced service load (if there is a higher risk), to support and correction outside of service provision (if the risk is high enough for the learner to be suspended from service activities) [6].

Despite a growing scholarly focus on remediation reform, current evidence regarding effective and efficient remediation practice remains limited [7, 8]. We know that we need to detect and correct deficits earlier in training programs, rather than later when deficits have compounded and the stakes are higher [9]. We also know that remediation usually works: learners who have been remediated are often indistinguishable from their non-remediated peers by the end of their training [10, 11]. And there can be problems associated with remediation. For instance, an emphasis on service rather than education can exacerbate the risk of failure for struggling learners [12–14]. Furthermore, many programs have difficulty placing learners on probation or dismissing them, often because of the fear of legal reprisal or faculty reluctance to judge the learners that they have been mentoring. Even when faculty are willing to report struggling learners, institutional barriers and a lack of common definitions and actions can make it difficult to identify and dismiss failing learners [15].

Remediation has typically been considered from the perspective of the individuals involved, either the remediator or the remediated, or both. However, remediation is intrinsically situated, it is always in context, and that context can shape and direct remediation practices both for good and ill. As Vignette 1 illustrates, competence is socially and contextually constructed [16]. Different training contexts afford different levels and forms of opportunity to succeed or fail as well as to remediate, and these contextual factors should also inform the design and conduct of remediation [17].

### Vignette 1: Sally’s story

*Sally Smith was a second-year internal medicine trainee. Although she had previously excelled in her medical training, this had obscured her poor clinical decision-making. When Sally needed to make decisions more independently for the first time, she over-focused on rare but potentially high-impact negative patient outcomes. Her preceptors noticed that she was slow to make decisions, over-used high-cost resources, and frequently called for consultations to rule out rare syndromes. They felt repeatedly frustrated and rebuffed by Sally’s perceived intransigence in doggedly pursuing workups, regardless of how straightforward the case.*

*Following complaints about her clinical work, Sally was told she needed to repeat her last rotation. Although aware of criticisms of her work, she did not fully understand its implications and her program director did not explain why she needed to repeat the rotation, other than she would need to improve her clinical performance and ‘read more.’ She was allowed to continue as a resident, but with the program director monitoring her work more closely than usual. The mixed messages and increased scrutiny unnerved her, making her more self-conscious. This exacerbated her tendency to overthink clinical decisions and as a result she spent less time supervising the interns. The repeated rotation did not go well, and Sally found herself facing dismissal from the program.*

*The culture of Sally’s training program emphasized independence and quick decision-making by preceptors and senior peers. Not surprisingly, trainees in this program quickly developed reputations as ‘good’ or ‘bad’ almost solely on that basis. An advisor suggested that Sally should transfer to another training program that would be a better ‘fit’ for her. Sally made the move and subsequently thrived in the new program, where she received regular mid-rotation and end of rotation feedback from her preceptors and felt respected by her peers and supervisors for her fund of knowledge. Through this process, she also gained awareness of her personal triggers toward high-intensity workups, developed a better sense of judicious use of resources, and garnered support for her increasing interest in subspecialty intensive care.*

Not only is remediation shaped by context, context is also shaped by remediation. The additional effort and potential distress associated with remediation, and the challenges in identifying specific learner deficits and effectively addressing them, typically position remediation as a burden, both practical and emotional. Remediation tends to be expensive and time-consuming, particularly for learners with multiple and/or severe challenges, significant learner debt, or other hardships. This can in turn skew the availability of resources and the commitment of educators. It can also establish a hidden curriculum of conflicted roles and responsibilities. We therefore need to consider remediation as a situated practice within broader societal, professional, and institutional systems.

## Towards a systems theory of remediation

**Fig. 1:**
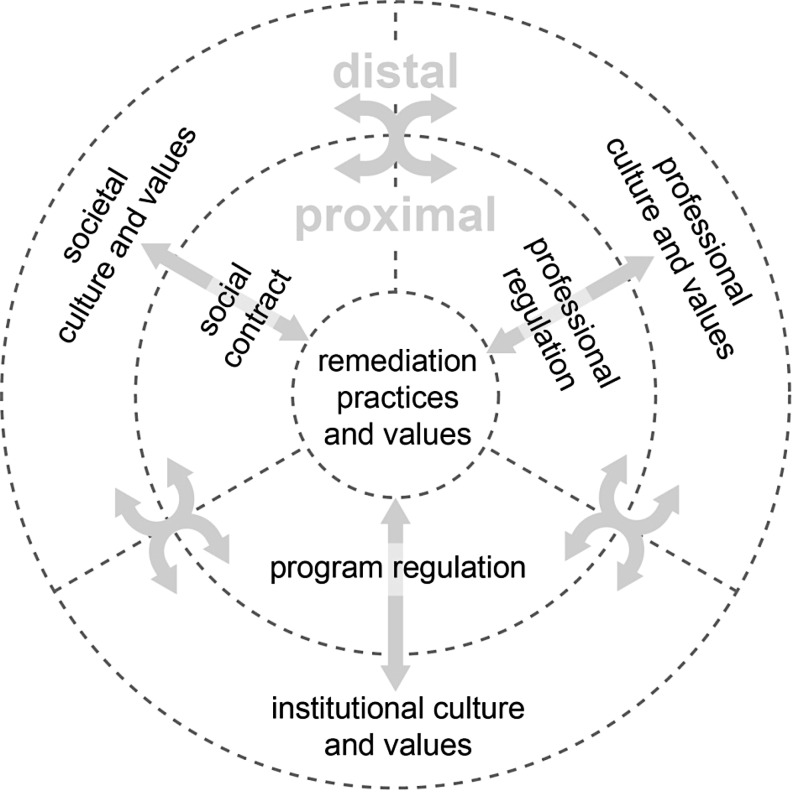
Multiple intersecting factors influence the practice and meaning of remediation: societal, professional, and institutional. These factors act at both proximal and distal levels and may be aligned to a greater or lesser extent, for instance in terms of their values, expectations, and authority. All of these influencing and shaping factors also interface with and influence each other. We can therefore understand remediation as an emergent set of practices that are shaped by the dynamic interactions between these contextual factors

We propose a system-level analysis of remediation, defined in terms of societal, professional, and institutional influences and accountabilities (Fig. 1) as a way of confirming the importance of the remediation process. We consider each of these elements in turn.

### Society

The social contract with medical education requires a consistent and effective production of trustworthy physicians who can deliver high quality and safe care, and who place this obligation above all others. In return, medical educators have significant autonomy to define and maintain their own standards of competence and the standards by which competence is assessed [18].

Although some values (such as basic professional standards) have international currency, other values (such as rewards, scope of practice, acceptability of risk and ambiguity, access of patients to healthcare services, and individual accountability and autonomy) can vary significantly between states, provinces, and countries (and sometimes even within them) [19]. These differences can inform the design of remediation programs in terms of what constitutes acceptable and unacceptable standards of practice, and how these processes are conducted and regulated. We should not therefore assume that remediation is (or should be) a standardized and normative undertaking, even in an increasingly globalized world. Indeed, social contexts will strongly influence the values and practices of remediation.

A growing number of medical schools have embraced increasing levels of accountability, both as a way to meet their social obligations and to protect the medical profession from litigation. In the same way that the medical profession has increased its attention to quality and safety in response to societal pressures about medical errors [20, 21], we argue that a similar conceptualization of remediation in medical education will justify an increase in trust in the profession. For instance, societal values (such as a patient’s protection from iatrogenic harm, or a disabled learner’s right to receive reasonable accommodations in the educational environment) should explicitly inform the extent to which learners should be given the opportunity to prove themselves as competent physicians. In addition, training programs must remember that litigation by failing learners rarely succeeds in the context of careful documentation [1], which should substantially obviate a defensive institutional stance towards failure.

### Profession

The regulation of the medical profession involves a complex interplay among professional societies, non-government organizations, and legislators. However, the extent to which the medical profession can self-regulate also varies greatly by jurisdiction [22, 23]. Indeed, although different organizations and agencies harbour different agendas, remediation is largely unregulated, except when it identifies critical flaws in learners’ characters. For instance, in the United States, remediation is mentioned sparingly in the accreditation standards for undergraduate and postgraduate medical education [24]. By contrast, in Canada, the College of Physicians and Surgeons of Ontario (CPSO) requires declaration of undergraduate and postgraduate remediation episodes for inclusion in their public physician register [25]. Moreover, although common practices in remediation have been proposed [5] and embraced by some [26, 27], assumptions that there are normative standards and practices for remediation across medical education, as a whole must balance with locally relevant and responsive practices.

### Institution

Given the limited societal and professional guidance around remediation, it falls to individual medical education programs to define the ways in which remediation is governed, resourced, and implemented. They also have to manage the many challenges associated with remediation. For instance, curriculum, assessment, and remediation can be or become misaligned in many ways. Lack of curricular flexibility, for example, may dictate that remediation cannot occur without significant disruption (in the absence of breaks between modules, intersessions between clerkships, and assessment weeks). This somewhat unforgiving structure, inexorably marching forward with little space for consolidation of learning, is particularly difficult for struggling learners and can increase the likelihood of ongoing failure. Ideally, individualizing medical education structures, as suggested by competency-based medical education [28], should maximize academic success rates. To do this effectively requires a robust understanding, both theoretically and pragmatically, about how best to tailor curricula.

A program of assessment that varies in structure (e. g., multiple choice tests, written essays, simulations, objective structured clinical evaluations), formative orientation, timing, or context, and judged holistically by trained individuals, can provide more effective scaffolding for weaker learners [5, 9, 29]. Furthermore, although it is often said that assessment drives learning, the reverse also appears true. For instance, minimally or tacitly assessed curricular elements (such as professionalism or systems-based practice) do not provide adequate early detection for struggling learners and could lead to a remediation ‘crisis’ once the learner moves into a context where that competence is required. An educational and assessment philosophy that is mastery-oriented on the other hand can support individual remediation rather than simply providing opportunities to fail [30].

Remediation practice also reflects institutional culture [31, 32]. We posit two dimensions of institutional cultures that impact remediation practices:

- A cultural dimension of *failure* arises from assumptions and beliefs regarding whether, when, and why individuals fail. This includes the thresholds between failure and normal variance in performance, what dimensions of suboptimal performance are more or less important, whether failure can and should be remediated, whether incidents of failure should be permanently recorded, and whether information about a struggling learner should be ‘fed forward’ or kept strictly confidential [33]. For a school to allow for any remediation at all reifies a collective belief that failure can be recovered from, and that there is a zone between passing and dismissal [33]. Mak-van der Vossen et al. have increased the willingness of medical school faculty to fail struggling students by enhancing their optimism about professionalism remediation with a systems-level intervention [34].
- A cultural dimension of *responsibility* is shaped by assumptions and beliefs regarding the mission of the school, its function, its goals, its accountabilities, and the way remediation aligns with these domains. For example, how much do a program’s responsibilities to society, its profession, its faculty, their patients, and other stakeholders balance with its responsibilities to their learners (as reflected in remediation practice)? How does this prioritization affect the design and practice of remediation? Is it more important to exclude ‘bad apples’ or to help those who misstep to recover and complete the program? What are learners’ responsibilities in remediation? To what extent should schools target their resources on the redemption of a few or on the successes of the many?

How institutions embody these cultural dimensions sends messages to all concerned and in turn informs how serious remediation is taken (both short and long term), who is responsible and how responsibility is expressed, and the place of remediation within medical education as a whole. We can therefore see remediation practice as occurring at the intersection between societal, professional and institutional systems and values (Fig. 1).

## Discussion

We are not the first to identify a need for theory and research to guide the complex, difficult decision-making process to remediate or dismiss learners from further training [5, 13, 35]. Our first step was to situate remediation within the broader systems of medical education and to clearly define the ways in which remediation differs from other practices and the implications of those differences [3]. In this paper, we have expanded our gaze to consider remediation in societal, professional and institutional contexts. This has allowed us to consider the shaping nature of these macro-level systems on remediation, and the ways in which the perspectives this provides can offer a foundation for remediation scholarship and innovation. For instance: Given that remediation draws upon a diverse set of philosophical, practical, and political concepts in what are often emotionally charged contexts, our analyses provide a compelling lens through which to view evidence, theory building, and application in medical education practice. Moreover, because remediation often marks the inflection point at which a learner is either judged to be capable of becoming a physician or not, it constitutes a liminal space between success and failure that sends complex messages to all concerned about their roles and obligations and the meaning of what is transacted there. As we have shown here, examining remediation from a systems perspective, rather than from the customary individual one, has a number of ramifications.

Paradoxes often arise in the context of remediation. Characteristics considered problematic in later stages of training may be undetectable early on, in part because they can emerge dynamically from the training process or could even be considered a strength in earlier phases of their training [36]. Vignette 1 epitomizes this possibility but is by no means the only route to remediation and potential failure. For instance, a learner with a single-minded, highly disciplined dedication to their own individual performance may rate highly with medical school admissions committees but show insurmountable difficulties when encountering complex clinical situations in residency training that require intricate interprofessional teamwork. Rather than assuming that improving admissions alone will reduce the need for subsequent remediation, we should instead acknowledge that flaws leading to remediation could actually emerge from medical education processes and systems. Similarly, although competency-based medical education includes mechanisms for earlier and more targeted detection of failing learners [27, 37], the costs of doing this may undermine the system-wide improvements that competency-based medical education is expected to bring.

### Vignette 2: Vin’s story

*Vin Bas was a third-year medical student in a 5-year undergraduate program. Although he had excelled at school, he found medical school hard going and had lost a lot of self-confidence as a result. Afraid to acknowledge his own shortcomings, Vin had become very defensive in his interactions with his teachers. Although Vin had sought to cover his weaknesses, he was unable to maintain this pretence and his performance over several clinical rotations had highlighted both his problems and his reluctance to face them.*

*Vin was at first very defensive when he met with his tutor to discuss the situation. However, the tutor took a different approach to the one Vin had been expecting in that she discussed what both patients and society as a whole expect from a practising physician and how that translated to medical training. Vin, who had previously thrived in a competitive academic environment, started to see things from a different perspective. He began to understand his moral obligation to be the best physician he can by acknowledging and learning from his shortcomings and being open to constructive criticism and correction. He also came to understand that it was not his right to be a physician that should shape his approach to learning, but society’s right to have the best physicians to provide its healthcare services.*

*Vin was changed by this experience. Not only did he become more open about his weaknesses, he was also more open to constructive criticism and was better able to make use of the supports that were available to help him become a good physician. By owning his shortcomings and being open to the help of others in his professional development, Vin successfully completed his training.*

The lack of a transparent and articulated remediation policy, combined with insufficient accountability for necessary invocation of the process, can produce a chilling effect on the peers of those who are underperforming and the faculty who interact with them. Tolerating ‘not yet’ competent or unprofessional learners diminishes the motivation of peers or faculty to intervene for fear of insufficient institutional support. This process also undermines the self-regulation that society grants the profession. In the worst case, implicitly or explicitly allowing some learners to struggle without adequate support diminishes the chances that others who need help will self-identify. A non-punitive, reliable and transparent remediation process supports a culture where all learners can hold themselves and each other to the very highest standards. This is illustrated in Vignette 2. If we accept that remediation is a natural, perhaps even desirable, component of medical education, then we must disconnect the tacit judgment about the effectiveness of the curriculum and faculty from the success of every individual learner. To that end, we need to work towards systems-level clarity and alignment of remediation principles in medical education. These principles should foster defensible and nuanced judgments about the current and future competence of individuals who will become physicians. These judgments need to first and foremost meet societal needs and values but should balance with professional and institutional needs and be fair and just to both learners and teachers. The application of these principles must be compassionate, discerning, and based on best educational theory and practice. Though Frankel (and others) [38–40] have provided case-based analyses of individual instances of remediating professionalism, there is much to learn about what constitutes effective remediation practices at the systems level in medical education. Nevertheless, a systems-level approach is required and, to that end, we make a number of recommendations:

- Remediation principles and practices should align with the medical education systems where they are situated. Currently, remediation tends to be an ‘outside’ activity, undertaken under duress and often unwillingly by all participants. This can encourage ‘rogue’ behaviour that undermines the process and can further separate the individual process from societal or professional needs. For instance, naming explicit requirements for initiation of remediation can facilitate a quality improvement process that all stakeholders, including learners, can embrace. This principle of ‘constructive alignment’ [41] lowers rather than raises barriers to remediation and ensures that the focus is on improvement.
- We must enable and support a continuum of remediation, ranging from individual improvements in day-to-day medical education, to highly structured episodes that may end with dismissal. As we have previously argued, the remediation continuum should encompass proportional management of different degrees and forms of remediation, including structure, equity, documentation, and closure [3].
- Remediation should be reframed from a matter of punishment and stigma to a form of training that many, if not most, will need and benefit from at some point. If failed remediation processes necessitate dismissal from training, compassionate systems would have already considered providing viable alternative career pathways or debt forgiveness.
- Optimally, institutions should develop a community of remediation practice, which contains the needed expertise. This should include all tutors, clinical preceptors, and supervisors who are able to recognize and refer learners who fall below a standard competence curve; a team of remediators, who use appreciative coaching techniques and the development of learning plans to support struggling learners and manage the remediation process; and an ultimate arbiter, represented by a program or course director, dean, etc., who consults with the other two groups to inform their final judgment about the outcomes of the remediation process.

We acknowledge a number of limitations to our arguments. We have positioned this paper in terms of a conceptual synthesis of current practices in remediation in medical education and the exposition of a systems perspective on reconfiguring these practices. Although individual components are evidence-based, we have argued for changes that are not yet in place and are therefore unavailable for review or critique. These proposals deserve testing through studies that consider a broad range of outcomes including impact on the individual learner, the relevant institutional stakeholders such as the medical school, clinical settings and the profession, and ultimately, the effect on individual patients and on the public’s health. We have also paid little attention to the costs, effectiveness, and sustainability of the systems we propose, and these too will need to be tested *in situ*.

## Conclusion

Remediation is part of a complex system of teaching and assessment that both shapes and is shaped by systems of medical education, as depicted in Fig. 1. Rather than an algorithmic or policy-bound response, remediation is socially constructed within educational ecosystems, and it sends messages about the nature of learning, support, assessment, regulation, process, identity, and so on, in and of a particular system of medical education. Remediation can thereby cast schools, programs, and faculty in a more or less confrontational and adversarial light. Therefore, a school’s culture of remediation can say much about its overall culture; indeed, it might be one of the signature acts that defines (or redefines) the institutional culture. Far from being an afterthought or an inconvenience, remediation should be a matter of seeing us at our best, or our worst. Like medical error, remediation could be pursued as an inevitable aspect of the complex system of medical training, not a failure of an individual. As is evident from the Quality Improvement movement, there is much more to be gained from anticipating the problem and preparing for it than hiding it. Remediating remediation practice in medical education has therefore never been more important or practical. However, as with all remediation, we may succeed or may fail in our efforts. The standard by which we will know whether we have succeeded flows from our ability to fully align remediation within medical education as a whole. Closer examination of the errors we make, the disappointments in our performances, and the remediation processes we may need to undergo requires courage on the individual, professional, and societal level to examine and benevolently hold our human foibles, consistently moving all toward improvement.
